# Frequency and Distribution of Broncho-Alveolar Fungi in Lung Diseases in Martinique

**DOI:** 10.3390/jcm12175480

**Published:** 2023-08-24

**Authors:** Moustapha Agossou, Jocelyn Inamo, Nelly Ahouansou, Marion Dufeal, Mathilde Provost, Elena Badaran, Adel Zouzou, Bérénice Awanou, Moustapha Dramé, Nicole Desbois-Nogard

**Affiliations:** 1 Department of Respiratory Medicine, CHU of Martinique, 97261 Fort-de-France, France; nelly.ahouansou@chu-martinique.fr (N.A.); marion.dufeal@chu-martinique.fr (M.D.); mathilde.provost@chu-martinique.fr (M.P.); elena.badaran@chu-martinique.fr (E.B.); adel.zouzou@chu-martinique.fr (A.Z.); sessito.awanou@chu-martinique.fr (B.A.); 2 Department of Cardiology, CHU of Martinique, 97261 Fort-de-France, France; jocelyn.inamo@chu-martinique.fr; 3 Department of Clinical Research and Innovation, CHU of Martinique, 97261 Fort-de-France, France; moustapha.drame@chu-martinique.fr; 4 EpiCliV Research Unit, Faculty of Medicine, University of the French West Indies, 97261 Fort-de-France, France; 5 Laboratory of Mycology, CHU of Martinique, 97261 Fort-de-France, France

**Keywords:** fungus, broncho-alveolar, lung disease, mold, yeast, bronchiectasis, pneumonia

## Abstract

The microbiota refers to all the microorganisms living in and on the human body; its fungal component is known as the mycobiota. The molecular component (mycobiome) has been linked to certain pulmonary diseases. Morphological fungal examination is still common practice and makes it possible to isolate fungi on direct examination or after sample culture. This study aimed to identify fungi via the genus colonising the respiratory tract in our environment and to evaluate the relationship between identified fungi and underlying diseases. We performed a retrospective study of patients who underwent bronchofiberoscopy and mycological analysis of fluid collected by broncho-alveolar lavage at our centre over a period of 5 years. During the study period, 1588 samples from 1547 patients were analysed (50.7% male, mean age 63.7 ± 14.8 years). Among the 1588 samples, 213 (13.4%) were positive on direct examination, and 1282 (80.8%) were positive after culture. The average number of species detected per sample was 1.4 ± 1.1. For patients with positive fungus, the median was two (ranging from one to seven). At least three fungal species were isolated in 14.4% of samples (17.9% of positive cultures), and at least two were isolated in 41.2% of samples (51.1% of positive cultures). Sterile mycelium was observed in 671 samples (42.28%), while *Candida* was identified in 607 samples (38.25%), and *Geotrichum* was identified in 271 samples (17.08%). Moulds were more frequently associated with bronchiectasis, while yeasts were associated with infectious pneumonia. Both moulds and yeasts were less frequent in diffuse interstitial lung disease, and yeast was less frequently present in chronic cough. Although overall, sterile mycelium and *Candida* were most frequently observed regardless of the underlying disease, there was nonetheless significant variability in the fungal genera between diseases. Fungal spores are highly prevalent in respiratory samples in Martinique. The species present in the samples varied according to the underlying respiratory disease.

## 1. Introduction

The microbiota and its fungal component, the mycobiota, have garnered increasing interest in recent years due to its established relationship with various diseases [1]. The microbiota refers to all the microorganisms that live in a given environment. The microbiome is the genetic composition of the microbiota and includes a bacterial component (bacterial microbiome), a fungal component (fungal microbiome or mycobiome), and a viral component (virome). Investigation of the microbiome and mycobiome has led to a greater understanding of the effects of various microorganisms on the human body. The gut has been the focus of the most intense research in this field, but the pulmonary microbiota is also widely investigated [2]. The pulmonary mycobiota refers to all the fungal species in the lungs, and the mycobiome is its genetic content. A growing body of evidence shows that the lung mycobiome is related to conditions such as asthma and impaired respiratory function, and it may impact the progression of lung diseases, such as cystic fibrosis and bronchiectasis [3]. The mycobiota varies widely according to the region and the local climate [4], and numerous studies have shown that the environment has a strong impact on the pulmonary mycobiota [5]. Indeed, the type and distribution of fungi varies across environments and climates. Moulds have long been known to play a role in asthma and allergies [6]. Several species have been shown to be associated with the prevalence of asthma and hospital admissions for asthma [7,8], with the most frequently incriminated species being *Aspergillus*, *Cladosporium*, *Alternaria,* and *Penicillium* [7,9]. Moulds may also be responsible for allergic bronchopulmonary mycosis and fungal rhinosinusitis, and their role in hypersensitivity diseases is also established [10]. Fungi can also cause invasive infections, such as invasive aspergillosis, pneumocystosis, histoplasmosis, and Penicillium, amongst others [10,11]. In addition, certain moulds are known or suspected to be toxic, although firm evidence is lacking for some alleged relationships between exposure to mould and toxicity [12,13].

In the literature, a limited number of studies describe the links between fungi, yeasts, and moulds and respiratory diseases, and all have been performed in temperate climates. To the best of our knowledge, no study to date has been undertaken in a tropical zone, or more specifically, the hot and humid Caribbean region. Martinique is a French island situated between the Caribbean Sea and the Atlantic Ocean. Its hot climate and high humidity are conducive to the development of various species of fungi, and many fungi and yeast species may be inhaled. Such inhaled microorganisms are often found in samples from the respiratory tract (e.g., expectoration, bronchial aspiration, and broncho-alveolar lavage). Apart from a few mould species, such as *Aspergillus*, whose pathogenic role is well-known, most fungi have little if any pathogenic potential in immunocompetent individuals. However, recent studies on the microbiome, and the mycobiome in particular, have shown that these species may play an important role in respiratory diseases. In daily practice, most centres do not routinely perform molecular analysis of the mycobiota, and conventional investigation consists of direct examination and culture of the sample. This approach makes it possible to isolate and identify certain fungi. However, the sensitivity of this approach is low, and cultures only isolate a very small proportion of the whole mycobiota, either because some spores are not viable and do not develop when cultured or due to inhibition or competition. In view of the growing body of literature pointing to the implication of the lung mycobiota in respiratory diseases, we believe that it is important to further investigate the impact of fungal colonisation on respiratory diseases.

The aim of this study was, therefore, to evaluate and identify the fungal species colonising the respiratory ecosystem and isolate them in culture and assess their potential association with underlying respiratory diseases.

## 2. Materials and Methods

### 2.1. Study Design and Eligibility Criteria

We performed a descriptive, analytical, retrospective study of samples harvested from 1 January 2018 to 31 December 2022.

The inclusion criteria were patients managed at the University Hospital of Martinique who had undergone bronchofiberoscopy with mycological examination of the bronchial liquid.

We excluded any patients who explicitly opposed the use of the data in their medical files for research purposes. For all patients, we retrieved socio-demographic data, the indication for bronchofiberoscopy, the definitive diagnosis, and the results of the mycological examination. 

### 2.2. Ethical Approval

The study was performed in accordance with the Declaration of Helsinki and received the approval of the Institutional Review Board of the University Hospitals of Martinique (number 2022/223).

The primary outcome was the prevalence of fungus in the respiratory sample obtained by bronchofiberoscopy. The secondary outcomes were the prevalence of fungus according to the underlying respiratory disease and the distribution of the fungal genera with or without species according to the underlying disease.

### 2.3. Underlying Respiratory Diseases

We considered the disease that justified performing the bronchofiberoscopy to be the main underlying disease in each patient. Patients were considered to have normal lungs when there was no justification for bronchofiberoscopy, i.e., respiratory symptoms, and normal thoracic imaging was normal.

### 2.4. Broncho-Alveolar Lavage Procedure

All patients with an indication for bronchofiberoscopy underwent bronchial or broncho-alveolar washing. During the study period, a total of 1547 patients underwent a mycological examination of the broncho-alveolar liquid (1588 samples) in our mycology laboratory.

For the lavage procedures, patients received local anaesthetic to the nose and oropharynx with Lidocaine 10%. The bronchofiberoscope was passed through the nose or mouth. The vocal cords were anaesthetised before the introduction of the bronchofiberoscope into the trachea and bronchi. Then, the saline solution was instilled and aspirated from the pathological area.

### 2.5. Mycological Examination of Bronchial Samples

Samples underwent direct examination and culture in compliance with the guidelines of the European Society of Clinical Microbiology and Infectious Diseases (ESCMID), the European Confederation of Medical Mycology (ECMC), and the European Respiratory Society (ERS) [14]. All mycological examinations were performed by a single biologist with expertise in the morphological classification of fungi using mass spectrometry for the identification of certain species, where appropriate.

– The diagnosis of yeasts was made by mass spectrometry.
– For filamentous fungi, the diagnosis was primarily mycological. Mass spectrometry was used to identify certain species that did not thrive on culture media.

Mass spectrometry was also used for the diagnosis of cryptic species, which have the same morphological appearance. Confirmation of the diagnosis was performed by an experienced biologist.

### 2.6. Statistical Analysis

A descriptive analysis was performed. Quantitative variables are described as the mean and standard deviation (SD), and categorical variables are described as numbers and percentages. Quantitative variables were compared using Student’s *t*-test, and categorical variables were compared using the chi-square or Fisher’s exact test, as appropriate. Bivariable analyses for the association between positive fungal culture and underlying pulmonary diseases were performed using binary logistic regression modelling. Statistical analyses were performed using SAS version 9.4, (SAS Institute, Inc., Cary, NC, USA). Tests were considered significant for *p*-values < 0.05.

## 3. Results

**Figure 1:**
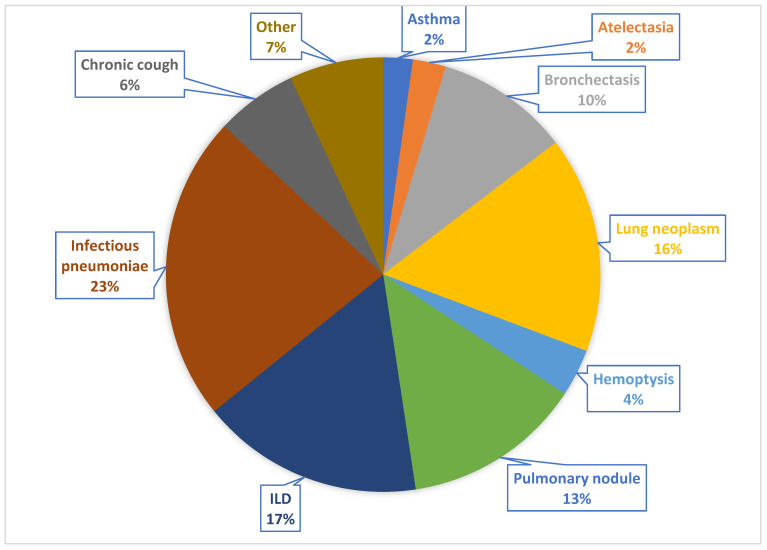
Proportion of patients with each underlying pulmonary disease. ILD: Interstitial lung disease.

During the study period, a total of 1547 patients underwent bronchofiberoscopy with 1588 procedures; 805 (50.7%) were men, and the mean age was 63.7 ± 14.8 years. The indications for bronchofiberoscopy are detailed in Figure 1.

Mycological examination:

Direct examination was positive in 213 patients (13.4%), and the culture was positive in 1282 samples (80.8%). The average number of species detected per sample was 1.4 ± 1.1 species. For patients with positive cultures, the median number of species was two (ranging from one to seven). At least three fungal species were isolated in 229 samples (14.4% of samples, 17.9% of positive cultures), and at least two species were isolated in 655 samples (41.2% of samples, 51.1% of positive cultures). 

The main fungal species identified are detailed in Table 1.

**Figure 2:**
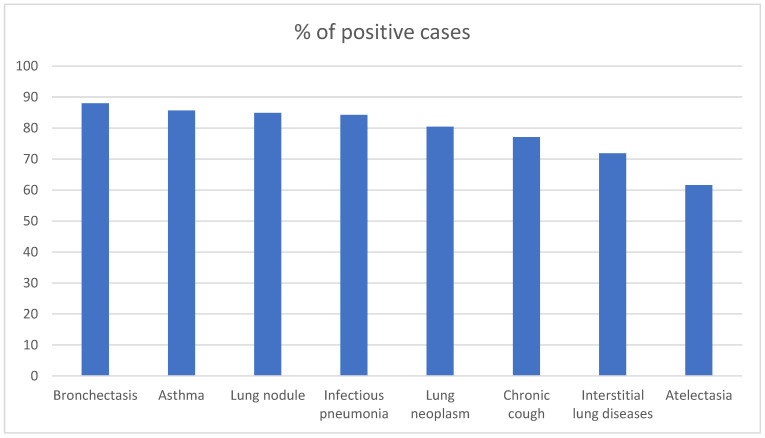
Proportion of positive culture for fungus according to the underlying lung disease.

Sterile mycelia were found in 671 samples (42.28%), while *Candida* was identified in 607 (38.25%), and *Geotrichum* was identified in 271 (17.08%). Figure 2 shows the prevalence of fungus according to the underlying diseases. 

The distribution of the fungal genera is displayed for each underlying disease in Supplementary Figures S1–S6.

In the bivariate analysis (Table 2), fungi were significantly less frequent in culture for atelectasis and diffuse interstitial lung disease.

Conversely, we observed a significantly increased presence of fungi in bronchiectasis. The association between the distribution of each fungal genus and the underlying diseases is shown in Table 3. 

Moulds were more frequent in bronchiectasis and chronic cough, whereas yeasts were more frequent in infectious pneumonia (Table 3). Moulds and yeasts were both less frequent in diffuse interstitial lung disease, while yeast was less frequent in chronic cough (Table 3). Sterile *Mycelium* and *Candida* were the most frequent for all diseases, but there was significant variability in fungal genera according to diseases (Figures S1–S6, Supplementary Material).

## 4. Discussion

In this study of over 1500 samples obtained through bronchofiberoscopy over a 5-year period, we found that direct mycological examination was positive in 13.4% of patients, and mycological culture was positive in 80.8% of patients. The fungal species found in the samples were predominantly yeasts, such as *Candida* and *Geotrichum*. The culture findings showed that 42% were sterile mycelia. The most difficult fungal species to identify are filamentous moulds, as previously reported by Desbois et al. [15]. The most abundant filamentous mould was *Penicillium*, followed by *Aspergillus*. Yeasts were predominantly represented by *Candida*, notably *C. albicans* and *Geotrichum* sp.

The lungs are the human organs that have the greatest exposure to the outside environment, and they are often affected by the air in the environment. Most of the filamentous fungi that we found in the bronchial lavage samples were identified in a review of fungal spores existing in Martinique performed by Desbois et al. [15] and in a 2022 report by a Martinique association investigating the quality of the air [16]. *Geotrichum* is a filamentous fungus that is classified as a yeast [17]. It is present in the ground as well as in water, plants, fruit, and dairy products. It penetrates via the digestive tract and, rarely, the respiratory route. The high prevalence observed in our population is likely due to substantial colonisation in our local environment, but *Geotrichum* was not identified in samples captured outdoors [15,16]. Possible explanations for the contamination include the inhalation of large amounts and significant aspiration from the digestive tract. 

*Candida* is the fungal component most widely implicated in disease [18]. *Candida albicans* is the most well-known in pathology, but non-albicans *Candida*, such as *Candida glabrata*, *Candida tropicalis*, and *Candida parapsilosis*, are also known to be pathogenic to humans [19], and they are all abundant in the environment in Martinique.

The lung mycobiota is the fungal component of the lung microbiota and appears to be related to chronic pulmonary diseases, such as asthma, chronic obstructive pulmonary disease, bronchiectasis, and cystic fibrosis [3,20]. A deleterious role of the pulmonary mycobiome has been established in all these diseases. Recently work has established an association between type 2 asthma and the lung mycobiome [21,22]. While metagenomic methods are more sensitive for characterising the lung mycobiome [3], culture remains a widely accessible technique that can be performed in specialised laboratories. The mycobiota could yield valuable insights about the mycobiome through its specificity, especially when the prevalence is as high as we observed in our study. 

Regarding the underlying diseases, we found a clear association between the presence of certain fungi and bronchiectasis. Environmental moulds were predominant, with sterile mycelia ranked first, followed by *Aspergillus* and *Penicillium*. Yeasts were mainly represented by *Candida* and *Geotrichum*. The majority of studies in the literature have found a predominance of *Aspergillus* and *Candida* in bronchiectasis [23,24]. The prevalence of *Aspergillus* in bronchiectasis reportedly varies between 6 to 58% in cystic fibrosis [24] while the prevalence of *Candida* was up to 45.2% in a Spanish study [23,25]. However, both these studies were performed in European countries, which do not have the same climate conditions as Martinique. 

Regarding asthma, we found the presence of fungi in 85% of patients with asthma, with a predominance of moulds, especially sterile mycelia, followed by *Penicillium* sp., *Aspergillus* sp., and *Geotrichum* sp. Vandenborght et al. previously reported a relationship between the indoor mycobiome and the clinical features of patients with severe asthma [26]. Furthermore, many indoor and outdoor fungal spores have been incriminated in the exacerbation of asthma and hospitalisations for asthma [7]. Finally, mould has also been implicated in declining respiratory function and a poorer response to bronchodilator treatment [27,28,29].

Previous investigations of the microbiome and idiopathic pulmonary fibrosis have suggested that the microbiome may have an injurious effect on the progression [30,31] and acute exacerbation [32] of diffuse interstitial lung disease. A similar impact was found in connective tissue-associated interstitial lung disease [33]. However, despite several studies on the role of the lung microbiome in the pathophysiology and progression of diffuse interstitial lung disease, there is a paucity of data regarding the impact of fungi on the disease and its natural course. Our data suggest that there is a lower abundance of fungi at the broncho-alveolar level in this context. Contrary to bronchial inflammation and the reduction in local immunity encountered in bronchiectasis, asthma, and, to a lesser degree, lung cancer, pulmonary infiltration by inflammatory cells in diffuse interstitial lung disease could be detrimental to the survival and multiplication of fungal spores. In a study in Finland, Laakkonen et al. reported that men with the highest mould and bacterial exposure had a reduced relative risk of lung cancer, whereas women with the highest mould and bacterial exposure were at a significantly increased risk of cervical cancer and lip cancer [34]. Certain fungi, such as *Blastomyces*, are reportedly associated with tumour tissue in lung cancer [35]. There is clearly a need for further research to determine the exact role of fungal colonisation in the genesis and progression of lung cancer.

In our study, patients with infectious pneumonia mostly had *Candida*, notably *C. albicans*. In a previous study, Krause et al. found no relationship between antibiotic treatment and colonisation with *Candida*, reporting that, compared with controls, *Candida* was part of the fungal microbiota in lower respiratory tract infections in patients without pneumonia, with and without antibiotic therapy [36].

This study is one of the few to date to investigate the prevalence of fungal spores in the lower respiratory tract and their relationship with underlying respiratory diseases. We showed high broncho-alveolar colonisation, even in those with no underlying respiratory disease. This underlines the strong impact of the environment on the respiratory system, which, although well-known, has been studied little in tropical areas. The large sample size confers strength to our analysis. However, our study also has some limitations; notably, the results are based on culture, which is not the most sensitive technique available nowadays for the identification of fungi. It is warranted to perform a more complete characterisation of the lung mycobiome in different lung diseases to evaluate the relationships between the fungi identified and each disease and the impact of fungal colonisation on disease progression.

## 5. Conclusions

In this study of over 1500 samples obtained through bronchofiberoscopy, we found a high prevalence of fungus in patients regardless of their underlying respiratory disease and even in those with no confirmed lung disease. There was a high proportion of environmental moulds, underlining their importance in lung disease. Certain conditions, such as bronchiectasis and infectious pneumonia, may be conducive to the presence of fungal spores, while others, such as diffuse interstitial lung disease and atelectasis, seem to be more conducive to the elimination of fungi. Further studies are needed to evaluate the effect of fungus on respiratory diseases in light of emerging knowledge about the mycobiome.

## Figures and Tables

**Table 1:** Distribution of the fungi isolated from 1588 bronchofiberoscopy samples.

Genera	N	%	Species	N	%
		Yeasts			
*Candida*	607	38.25	*Albicans*	537	33.84
			*Tropicalis*	62	3.91
			*Glabrata*	37	3.33
			Others	78	4.91
*Geotrichum*	271	17.08			
		Moulds			
*Aspergillus*	186	11.72	Section *Nigri*	104	6.55
			Section *Fumigatus*	35	2.21
			Others	60	3.78
*Penicillium* sp.	187	11.78			
*Cladosporium* sp.	43	2.71			
Sterile mycelium	671	42.28			
Other moulds	169	10.64			

**Table 2:** Association between presence of fungi on culture and underlying diseases.

Underlying Diseases	N	Positive Culture	%	*p*
All	1587	1282	80.8	
Atelectasia	39	24	61.5	0.007
Infectious pneumonia	362	305	84.3	0.06
Interstitial lung diseases	263	189	71.9	0.0001
Bronchiectasis	158	139	88	0.02
Lung cancer	256	206	80.5	1
Lung nodule	212	180	84.9	0.11
Chronic cough	96	74	77.1	0.4
Asthma	35	30	85.7	0.6

Missing data = 1.

**Table 3:** Association between mould and yeast distribution and the underlying respiratory disease.

Clinical Context (N)	Moulds		Yeasts	
	Positive Cases (%)	*p*	Positive Cases (%)	*p*
Atelectasia (39)	20 (51.3)	0.48	20 (51.3)	0.99
Bronchiectasis (158)	108 (68.4)	0.006	88 (55.7)	0.32
Asthma (35)	24 (68.6)	0.27	15 (43.9)	0.38
Diffuse interstitial lung disease (263)	134 (51)	0.01	111 (42.2)	0.001
Infectious pneumonia (362)	199 (55)	0.19	217 (59.9)	<0.001
Chronic cough (96)	67 (70)	0.02	27 (28.1)	<0.0001
Lung cancer (256)	142 (55.5)	0.45	143 (55.9)	0.15
Lung nodules (212)	133 (62.7)	0.15	80 (37.7)	0.99
Normal lungs (35)	25 (71.4)	0.14	15 (42.9)	0.38

## Data Availability

The datasets generated and/or analysed in the current study are available from the corresponding author upon reasonable request.

## Supplementary Materials

The following supporting information can be downloaded at: https://www.mdpi.com/article/10.3390/jcm12175480/s1, Supplementary Figure S1: Distribution of fungal genera in samples from patients with normal lungs. Supplementary Figure S2: Distribution of fungal genera in samples from patients with asthma. Supplementary Figure S3: Distribution of fungal genera in samples from patients with bronchiectasis. Supplementary Figure S4: Distribution of fungal genera in samples from patients with lung cancer. Supplementary Figure S5: Distribution of fungal genera in samples from patients with infectious pneumonia. Supplementary Figure S6: Distribution of fungal genera in samples from patients with diffuse interstitial lung disease.
