# Supplementary material for: Frequency and Distribution of Broncho-Alveolar Fungi in Lung Diseases in Martinique
Source: J Clin Med. 2023 Aug 24;12(17):5480. doi: 10.3390/jcm12175480 (PMC10488106; doi:10.3390/jcm12175480)

Supplementary Material

Supplementary Figure Legends

Supplementary Figure S1

Distribution of fungal genera in samples from patients with normal lungs.

Supplementary Figure S2

Distribution of fungal genera in samples from patients with asthma.

Supplementary Figure S3

Distribution of fungal genera in samples from patients with bronchiectasis.

Supplementary Figure S4

Distribution of fungal genera in samples from patients with lung cancer.

Supplementary Figure S5

Distribution of fungal genera in samples from patients with infectious pneumonia.

Supplementary Figure S6

Distribution of fungal genera in samples from patients with diffuse interstitial lung disease.

Supplementary Figure S1

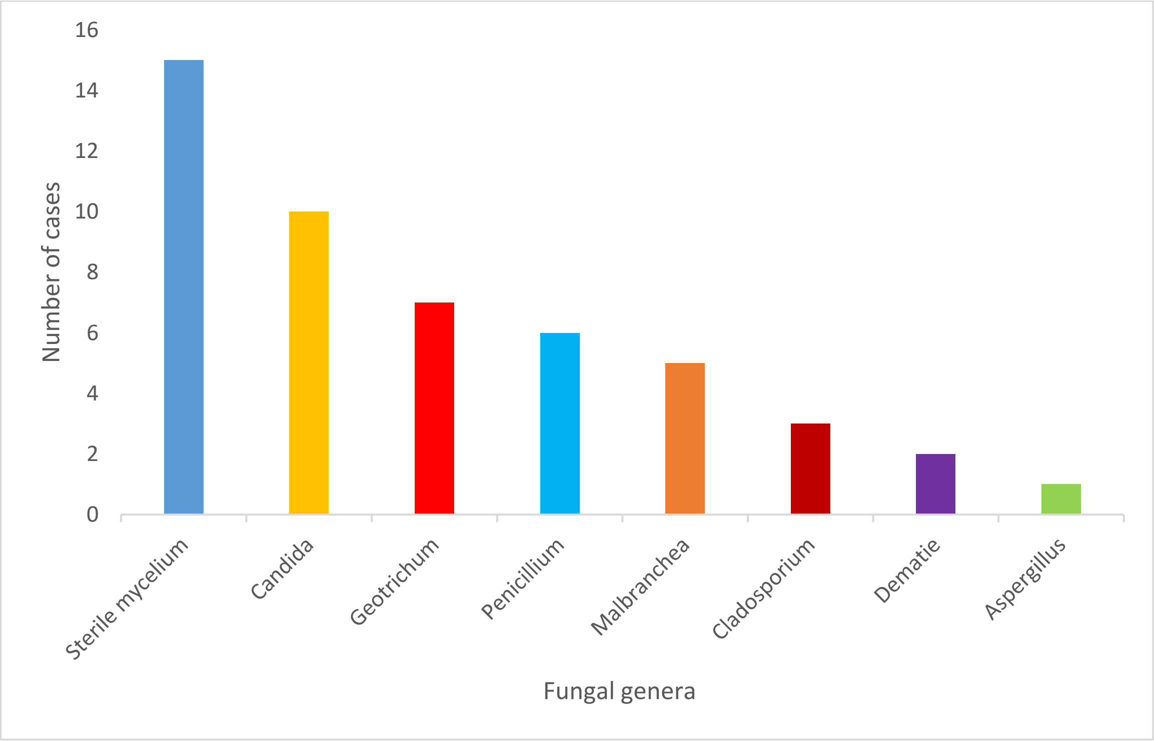

Supplementary Figure S2

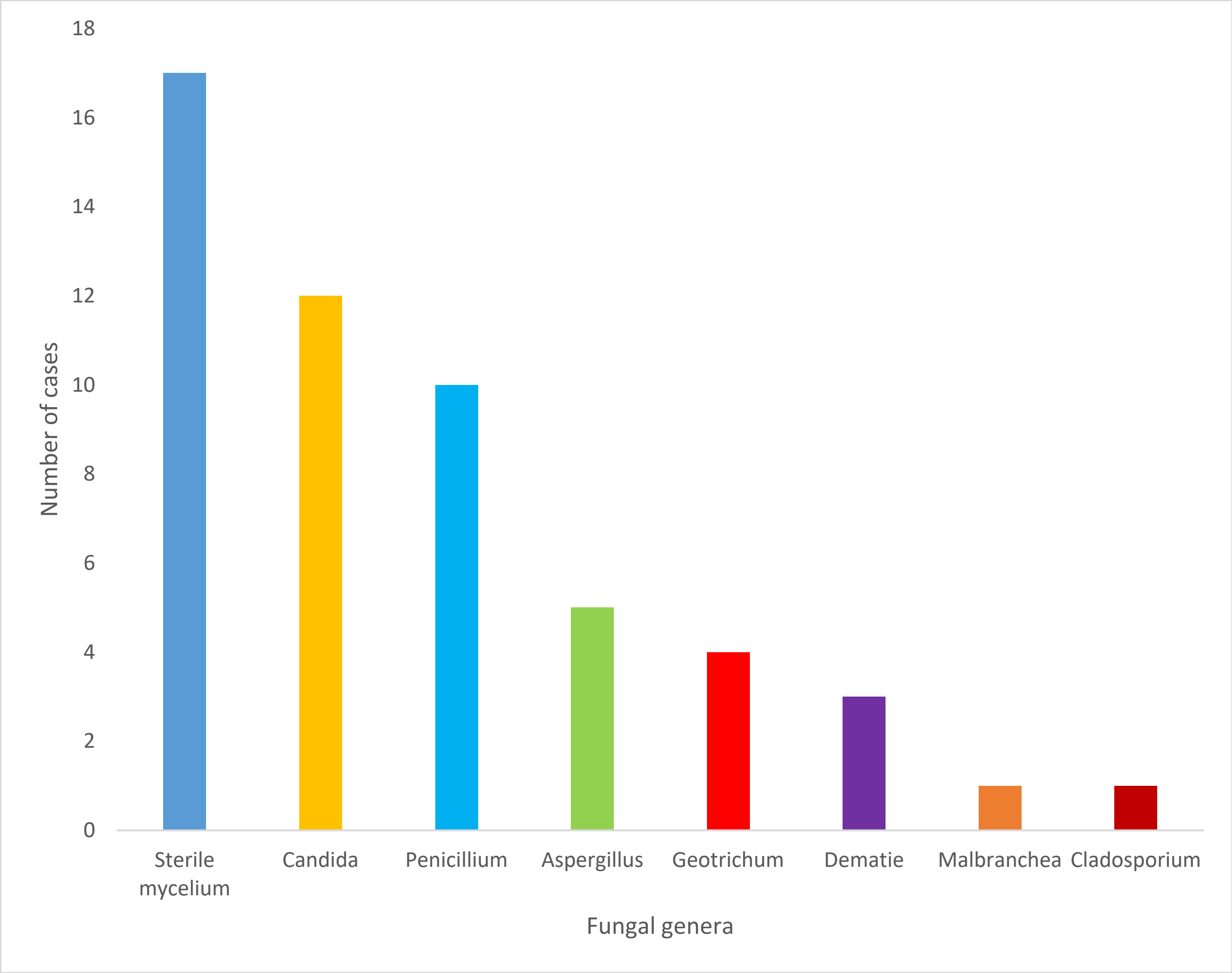

Supplementary Figure S3

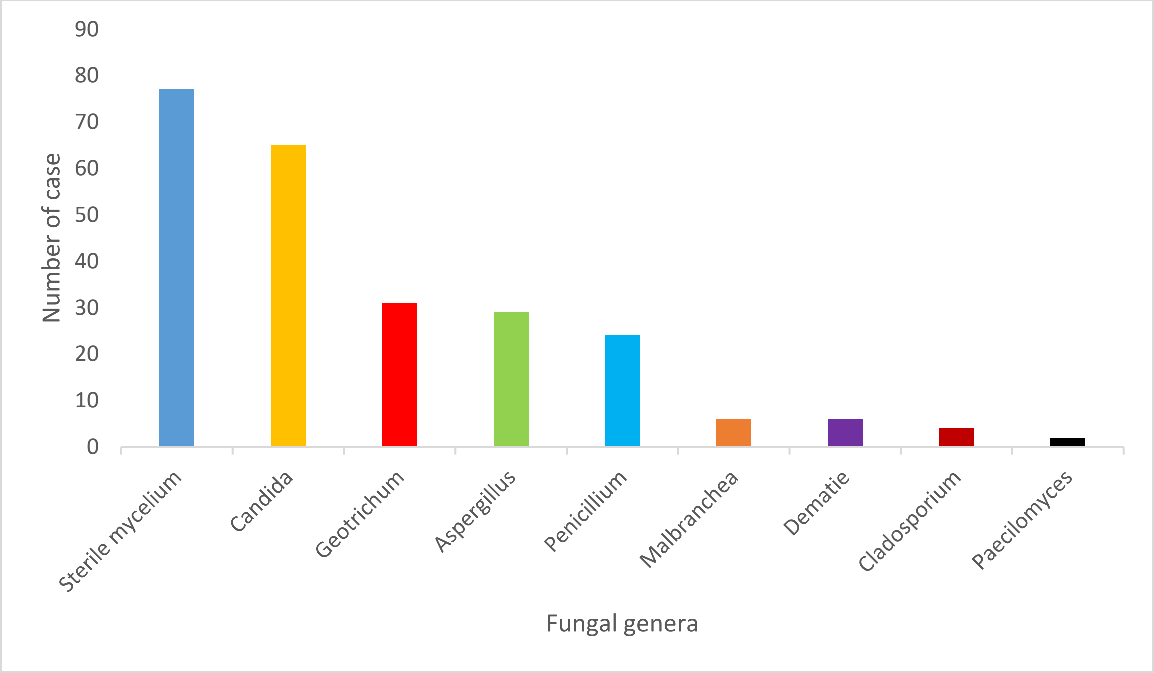

Supplementary Figure S4

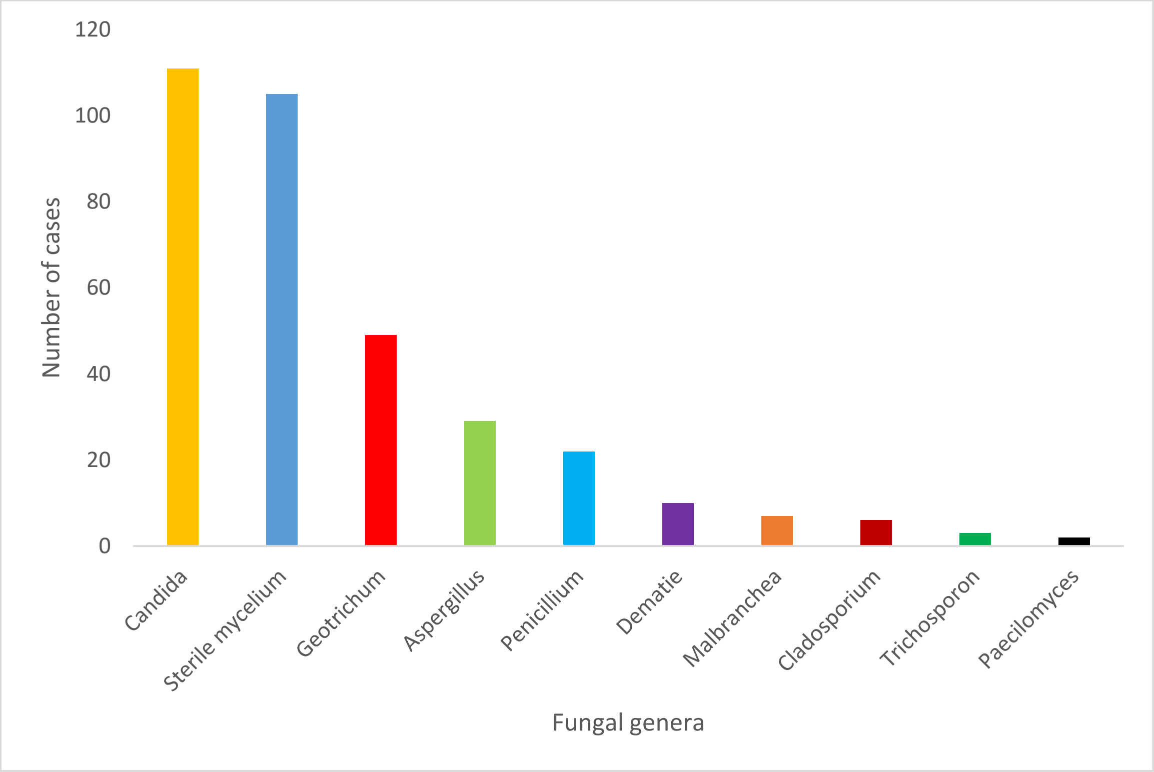

Supplementary Figure S5

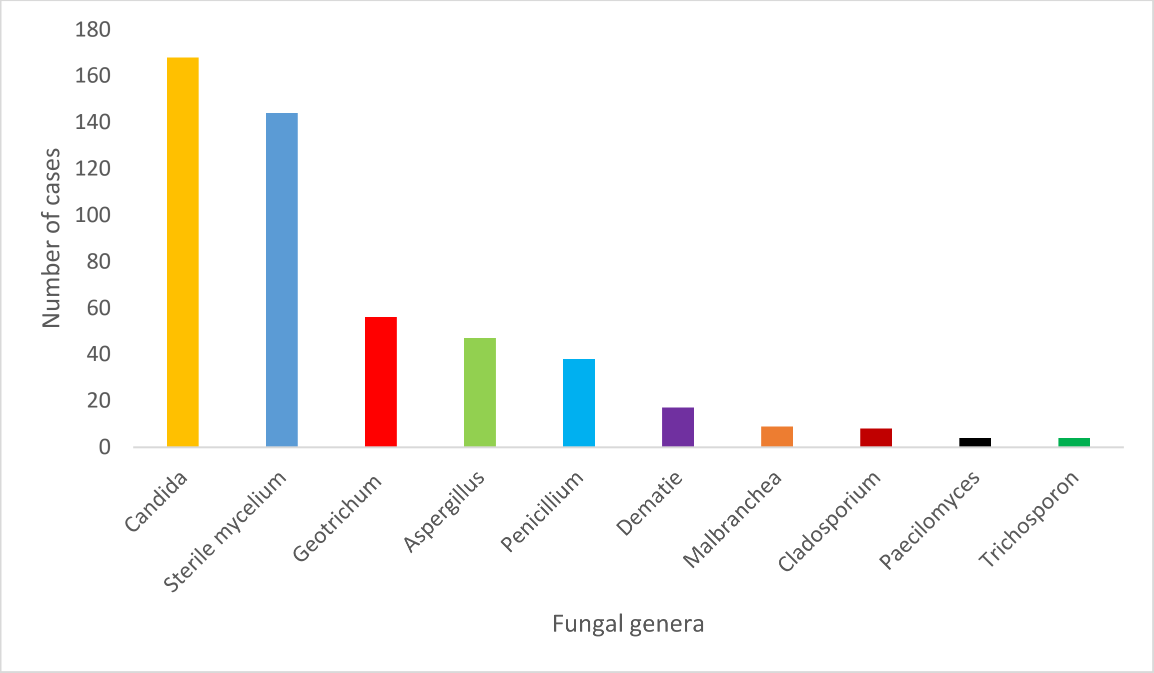

Supplementary Figure S6

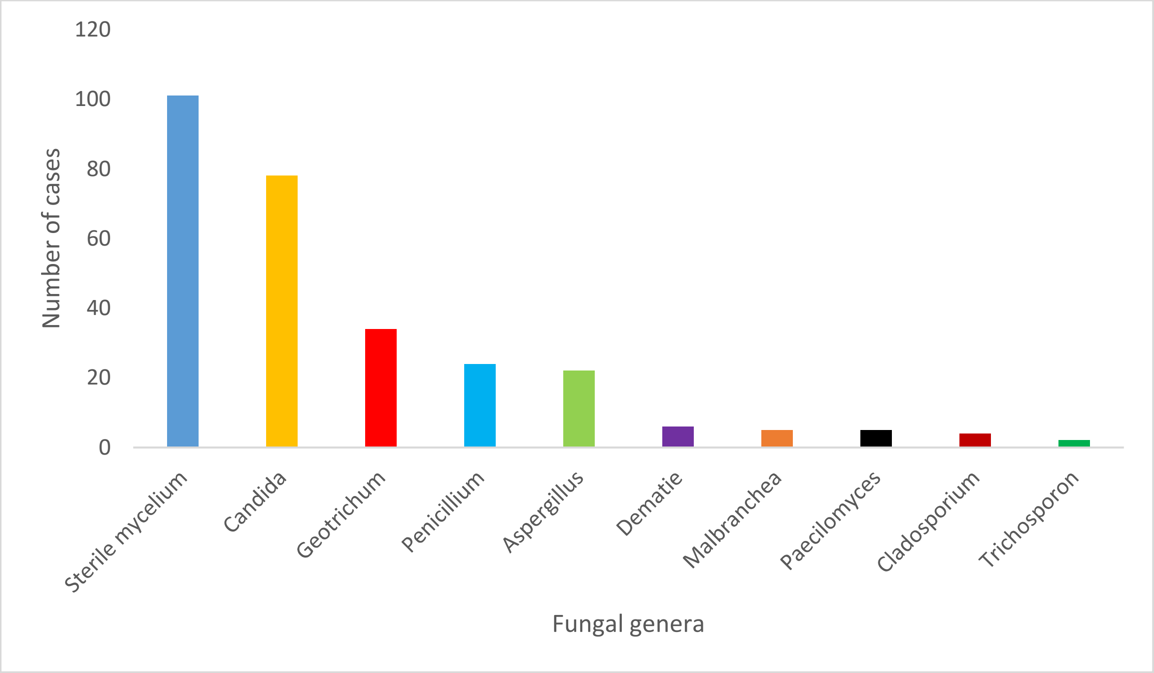

Supplement: Supplementary file 1 [file jcm-12-05480-s001.zip › jcm-2514322-supplementary.pdf]
